# Chemical Composition and Biological Activities of Tunisian *Ziziphus lotus* Extracts: Evaluation of Drying Effect, Solvent Extraction, and Extracted Plant Parts

**DOI:** 10.3390/plants10122651

**Published:** 2021-12-02

**Authors:** Touka Letaief, Stefania Garzoli, Valentina Laghezza Masci, Jamel Mejri, Manef Abderrabba, Antonio Tiezzi, Elisa Ovidi

**Affiliations:** 1 Laboratory of Materials Molecules and Applications (LMMA), IPEST, University of Carthage, BP 51, La Marsa 2070, Tunisia; touka.letaief@gmail.com (T.L.); jmejri2001@yahoo.fr (J.M.); manef.abderrabba@ipest.rnu.tn (M.A.); 2 National Agronomic Institute of Tunisia (INAT), University of Carthage, Mahrajene 1082, Tunisia; 3 Department for the Innovation in Biological, Agrofood and Forestal Systems, Tuscia University, 01100 Viterbo, Italy; laghezzamasci@unitus.it (V.L.M.); antoniot@unitus.it (A.T.); eovidi@unitus.it (E.O.); 4 Department of Drug Chemistry and Technology, Sapienza University, 00185 Rome, Italy

**Keywords:** *Ziziphus lotus*, phenolics, antioxidant activity, SH-SY5Y cell line, chromatography

## Abstract

The Tunisian *Ziziphus lotus* plant was investigated to determine its phytoconstituents and evaluate its biological activities. In particular, the GC/MS technique was used to describe the chemical composition of *Z. lotus* active extracts and fractions. Among the obtained extracts, the yields of the dried root methanolic extract (29.80%) and the fruit aqueous extract (48.00%) were the highest ones. The dried root methanolic extract exhibited the highest amount in the total phenolics (186.44 ± 0.26 mg GAE/g DW), total flavonoids (102.50 ± 3.53 mg QE/g DW), and tannins (60.714 ± 2.2 mg catechin/g DW). The root aqueous extracts revealed the highest antioxidant activity with an IC_50_ of 8.96 ± 0.38 mg/L and 16.46 ± 0.60 mg/L for the ABTS^•^^+^ and DPPH• assays, respectively. The total antioxidant capacity was accorded to the methanolic extract of the dried roots with a value of 304.07 ± 1.11 µg AAE/mg. The drying process was found to improve the qualitative and quantitative properties of the *Z. lotus* extracts. The evaluation of the cytotoxic activity against the SH-SY5Y cell line was carried out using MTT assay. The petroleum ether and dichloromethane extracts of the dried roots showed relevant cytotoxic activities. The thin layer chromatography and the GC-MS/GC-FID analysis led to the identification of the 13-epimanool as a potent cytotoxic compound.

## 1. Introduction

Medicinal and aromatic plants represent an inexhaustible reservoir of secondary metabolites. These compounds do not play an essential role in plant growth, like the primary metabolites do, but rather serve to enable the plant to cope with extreme environmental conditions (drought, oxidative stress, pests, etc.), allowing better interaction between the plant and its surrounding environment [1]. Numerous classes of secondary metabolites exist according to their chemical structure, route of biosynthesis and solubility in water and organic solvents [2]. In order to explore such metabolites, an adequate extraction and isolation of the bioactive compounds, the screening of phytoconstituents, and the evaluation of their potential activities are needed [3]. Among the identified plants, only around 20% were studied in pharmaceutical and medicinal research [4], whereas many other plants are still not thoroughly explored [5]. In this context, the shrub *Z. lotus*, commonly named ‘Sedra’, is the subject of many current laboratory investigations. This plant belongs to the Rhamnaceae family which includes around 550 species spread over around 45 genera [6,7]. Being both a tropical and a subtropical plant, *Z. lotus* is commonly present in arid and semi-arid regions [8]. In Tunisia, this xerophytic plant can be found in the sand dunes of Saharan regions as well as in the arid and semi-arid zones where it occupies different types of soils [8,9]. *Z. lotus* is dormant from October to March and its fruits are harvested during the summer [10]. It forms clumps of a few meters in diameter and 2 to 5 m in height. Its thorny stems possess small deciduous leaves and tasty fruits called ‘Nbeg’ [6,8,11].

*Z. lotus* is a versatile shrub that is highly interesting especially for the inhabitants of dry areas [12]. It has been used in traditional medicine for the treatment of many diseases. In fact, it is used to treat diarrhea and regulate blood sugar levels [11], while its tonic febrifuge fruit is used for the dissolution of kidney stones [13]. Thanks to its emollient properties, the paste of this fruit has been used as an excellent pectoral remedy [6]; furthermore, it has been employed for the treatment of hazardous viral diseases like measles and smallpox [6].

Many findings in the research confirmed these traditional uses. Borgi et al. [14] outlined the anti-inflammatory, analgesic and antispasmodic activities of *Z. lotus* extracts. The aqueous extracts obtained from different parts of *Z. lotus* proved their effects as cytotoxic agents against T-cells, the major cause of autoimmune diseases [15]. Furthermore, *Z. lotus* extracts obtained by different solvents showed excellent antifungal activity against nine strains of pathogenic fungi [16]. Such endowments reflect the richness of *Z. lotus* in many active compounds, notably flavonoids and tannins [14], cyclopeptide alkaloids such as lotusine A and lotusine D [17], and vitamins (A, C, E) [15].

The aim of this work is to further investigate *Z. lotus* extract potencies. After the preparation of extracts from the dried and fresh parts (roots, leaves, fruits) of this plant by using different solvents, a screening of the phytoconstituents was carried out. The evaluation of the antioxidant activity was performed by ABTS^•^^+^, DPPH•, and TAC assays. In addition, the cytotoxic activity against the SH-SY5Y cell line was investigated. The extract showing a potent effect against this cancer cell line was subjected to GC-MS analysis.

## 2. Results

### 2.1. Extraction Yields

The yields of *Z. lotus* (root, leaf, and fruit) extracts obtained by different solvents were reported in Table 1. Extraction yields were between 0.50% and 48%.

### 2.2. Chemical Compositions of Z. lotus Extracts

Total phenolic content (TPC), total flavonoid content (TFC) and tannins of *Z. lotus* extracts were summarized in Table 2. Total phenolic content (TPC) of *Z. lotus* extracts, expressed as Gallic Acid Equivalent, ranged between 11.16 ± 0.13 and 186.44 ± 0.26 mg GAE/g DW. Among these phenolic compounds, flavonoids and tannins reached a maximum of 102.50 ± 3.53 mg QE/g DW and 60.71 ± 2.20 mg CE /g DW, respectively. These highest values were assigned to the methanolic extract of the roots.

### 2.3. Antioxidant Activity 

The antioxidant potential of *Z. lotus* extracts was evaluated by using 2,2-azinobis (3-ethylbenzothiazoline-6-sulfonic acid) (ABTS^•^^+^), 2,2-diphenyl-1-picrylhydrazyl (DPPH•) and Total Antioxidant Capacity (TAC) methods. Table 3 reported the IC_50_ values. The ABTS^•^^+^ colorimetric assay determined IC_50_ values ranging from 8.96 ± 0.38 to 136.58 ± 0.41 mg/L for the root extracts and from 23.48 ± 0.63 to 249.37 ± 1.26 mg/L for the leaf extracts. The fruit extracts showed a low activity compared to the other part of the plants. The ME of the roots, with a concentration of 18.03 ± 0.61 mg/L, allowed a 50% inhibition of the free radical DPPH•. This methanolic extract possessed the potent total antioxidant capacity equivalent to 304.07 ± 1.11 mg ascorbic acid per mg of extract.

### 2.4. Drying Effect on Phytochemical Composition and Antioxidant Activity

**Figure 1:**
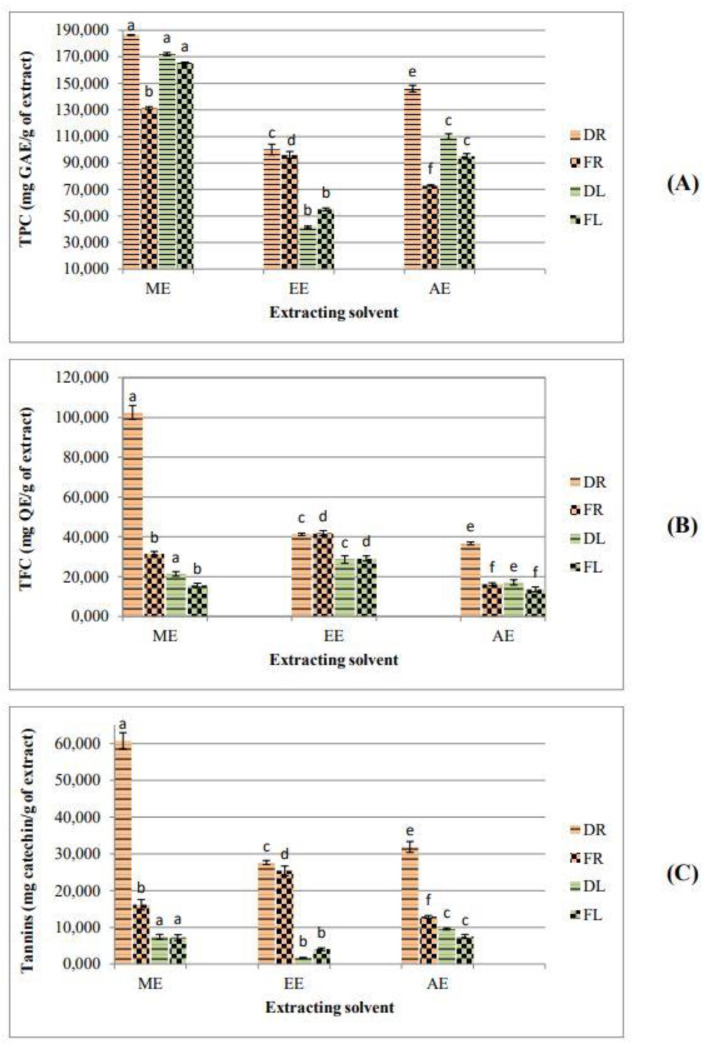
Drying effect on phytochemical composition of *Z. lotus* extracts. (**A**) Total phenolic content; (**B**) Total flavonoid content; (**C**) Tannin content. DR: Dried Roots; FR: Fresh Roots; DL: Dried Leaves; FL: Fresh Leaves. Histograms with the same colour marked with different letter were significantly different (*p* < 0.05).

Quantitative evaluation of total TPC, TFC and tannins in *Z. lotus* extracts from the dried and fresh roots (DR and FR, respectively) and leaves (DL and FL, respectively) were summarized in Figure 1. Root extracts remained the highest in phytoconstituents. For all the extracting solvents, the shade-dried samples of *Z. lotus* roots presented higher values of phytochemical compounds in terms of phenolics, flavonoids, and tannins, than the fresh samples. As regards the leaves, the drying did not show a considerable effect on this chemical composition. The phosphomolybdenum assay for the total antioxidant capacity evaluation, confirmed the potent antioxidant capacity of the root extracts.

**Figure 2:**
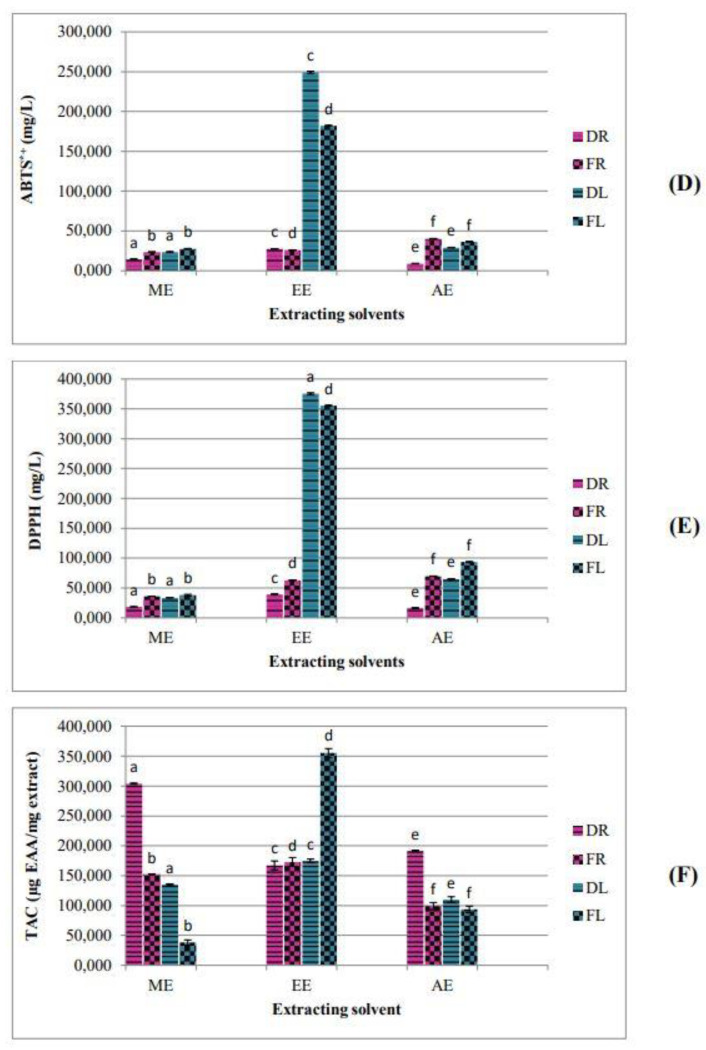
Drying effect on antioxidant activities of *Z. lotus* extracts. (**D**) ABTS^•^^+^ scavenging activity; (**E**) DPPH• scavenging activity; (**F**) Total antioxidant capacity. DR: Dried Roots; FR: Fresh Roots; DL: Dried Leaves; FL: Fresh Leaves. Histograms with the same color marked with different letter were significantly different (*p* < 0.05).

For both the ABTS^•^^+^ and DPPH• assays, the samples presenting the lowest IC_50_ values are the potent ones. Hence, the extracts of the dried roots are more efficient than the extracts of the fresh roots. The ethanolic extracts of the leaves (dried and fresh) show the lowest antioxidant activity (Figure 2).

### 2.5. Chemical Characterization of the Dried Root Petroleum Ether and Dichloromethane Extracts: GC-MS Analysis and Thin Layer Chromatography

The analysis of *Z. lotus* samples was carried out using a TurboMass Clarus 500 GC-MS/GC-FID. The main compound was n-hexadecanoic acid (90.6%) and Tetradecanoic acid, ethyl ester (72.8%), for the dried root petroleum ether extract (DR-PEE) and dried root dichloromethane extract (DR-DE), respectively. The compound 13-epimanool was a common compound of both petroleum ether and dichloromethane root extract fractions.

### 2.6. Cytotoxic Activity

The evaluation of the cytotoxic activity against the SH-SY5Y cell line was carried out by MTT assay. After 24 h of treatment, these human neuroblast cells revealed sensitive to the DR-PEE and DR-DE with an IC_50_ of 184.413 ± 4.77 and 16.148 ± 0.93. Increasing the treatment time to 48 h, improved considerably the cytotoxic potential of the extracts. The strongest activity (7.341 ± 1.98 µg/mL) was obtained by the DR-DE after 48 h of treatment (Table 4).

## 3. Discussion

The yield of the extraction depended on the solvent type and the extracted part of *Z. lotus*. The yield of methanolic extracts of roots and leaves resulted in 29.80% and 15.10%, respectively; and the water extract of the fruit allowed an extraction yield of 48.00%. The yields of the petroleum ether and dichloromethane extracts of the fruit were very low (data not shown). As suggested by Climati et al. [18], higher yields of the polar extracts (methanol, ethanol and aqueous) reflected the richness of *Z. lotus* samples in polar compounds.

The polar extracts of the roots and leaves showed the highest amount in phenolics ranging from 41.69 ± 0.70 mg GAE/g DW to 186.44 ± 0.26 mg GAE/g DW, which encompassed flavonoids, tannins, and other chemical compounds. This is probably due to the efficient interaction between the polar sites of the antioxidant compounds and the polar solvents (aqueous, methanolic, and ethanolic). The TPC of polar extracts was significantly (*p* < 0.05) affected by the extracting solvent. The methanolic extracts of roots and leaves presented the highest TPC amount; and the aqueous extract of the fruit was the richest one in phenolics. These TPC amounts might be affected by the interference of some other compounds that reduce the Folin-Ciocalteu reagent [19]. On the other hand, petroleum ether and dichloromethane extracts contained the lowest amounts of phytochemical compounds without a significant difference between the two extracting solvents.

The TFC values were significantly (*p* < 0.05) affected by the extracting solvent. For both roots and leaves, the highest values were assigned to the ME and EE regarding the PEE, DE, and AE. The tannin contents of roots and leaves ranged from 6.88 ± 1.41 to 60.71 ± 2.2 mg CE/g DW and from 1.66 ± 0.09 to 9.54 ± 0.26 mg CE /g DW, respectively. The ME and AE of the fruits presented the lowest tannin content with no significant difference between the two extracting solvents.

Concerning tissue phytochemical compositions as shown in Table 2, the TPC, TFC, and tannin contents varied significantly (*p*< 0.05) regarding the plant part. The root barks of *Z. lotus* presented the highest amount of phenolics (186.44 ± 0.26 mg GAE/g DW), flavonoids (102.50 ± 3.53 mg QE/g DW), and tannins (60.71 ± 2.20 mg CE/g DW); and these values were higher than those reported by Ghalem et al. [20]: TPC was 20.09 mg/g DW, TFC 0.02 mg/g, and tannins 1.56 mg/g DW, for the same species extracted with 70% acetone. In addition to the extracting solvent, other factors such as the harvesting period, the plant origin, and environmental conditions could explain this variability [21]. The leaf phenolics amount ranged from 11.16 ± 0.45 to 171.99 ± 1.14 mg GAE/g DW. The ME of the dried leaves was the richest one. These phenolic amounts were higher than those determined by Elaloui et al. [21] (21.98 mg GAE/g DW) and by Guirado et al. [9] (9.498 mg GAE/g DW). Taken all together, our findings confirmed the potent methanol capacity to extract the phytochemical compounds from *Ziziphus* leaves [21].

The flavonoid and tannin values of the leaves respectively varied between 3.06 ± 0.12 to 28.54 ± 1.89 mg QE/g DW and 1.66 ± 0.09 to 9.54 ± 0.26 mg CE/g DW. As demonstrated by Maraghni et al. [8], the plant endures extreme environmental conditions by the synthesis of flavonoids and tannins. This could clearly explain the richness in secondary metabolites of our samples, collected from a desert region in southern Tunisia.

The AE of the fruit was rich in phenolics (82.12 ± 1.70 mg GAE/g DW), flavonoids (13.40 ± 0.72 mg QE/g DW), and tannins (1.02 ± 0.10 mg CE/g DW). Samples investigated by Khouchlaa et al. [13] showed higher values of these bioactive compounds (phenolics 285.19 mg GAE/mg, flavonoids 2.66 mg QE/mg).

IC_50_ values obtained by ABTS^•^^+^ assay were significantly (*p* < 0.05) affected by the solvent used as well as the plant part. For almost all the extracting solvents, *Z. lotus* root extracts showed higher antioxidant activity than the leaves and fruits. The most active samples were AE (8.96 ± 0.38 mg/L) followed by the ME (14.31±0.13 mg/L) and the PEE (14.76 ± 0.02 mg/L) of the roots. For the leaves, the ME showed the best IC_50_ value (23.48 ± 0.63 mg/L). This IC_50_ value was higher than the value (3.82 mg/L) determined by Abderrahim et al. [22], studying the Algerian *Z. lotus* methanolic extract of the leaves. This variation could be due to the plant origin and the extracting method. In fact, in our study the leaves were successively extracted with solvents of increasing polarity, whereas in the mentioned study the ME was obtained by a direct extraction of the leaves by methanol.

Confirming the ABTS^•^^+^ assay, the highest DPPH• antioxidant levels were detected in root extracts. The extraction solvent and the plant part significantly (*p* < 0.05) influenced DPPH• IC_50_ values. Both the AE and the ME of this plant part were ranked as potent extracts with an IC_50_ of 16.46 ± 0.60 mg/L and 18.03 ± 0.61 mg/L, respectively. The ME of the leaves expressed the highest value among this plant organ extracts (33.66 ± 0.11 mg/L), almost as high as the one (28.19 mg/L) reported by Abderrahim et al. [22]

In both ABTS^•^^+^ and DDPH• assays, the polar extracts (ME, EE, AE) showed higher quenching behaviour than the non-polar extracts (PEE, DE). This could be due to the richness of polar extracts in phenolics. The ABTS^•^^+^ and DPPH reducing ability followed the same trend for root and leaf extracts (AE > ME > EE) and (ME > AE > EE), respectively. However, in the DPPH• assay IC_50_ values were higher; hence, the scavenging activity of the samples for this radical is lower. These results agree with many previous studies such as the investigations on the aqueous, acetone, and ethanol extracts of *Ziziphus mucronata* Willd. subsp.*mucronata* Willd [23]. Consequently, *Z. lotus* extracts react better with the ABTS^•^^+^ assay which is based on rapid electron transfer reactions.

*Z. lotus* total antioxidant capacities (TAC) expressed in Equivalent of Ascorbic Acid (AAE/mg) showed a significant (*p* < 0.05) variability according to the solvent and tissue. Extracts from polar solvents as the ME and AE of the roots and the EE of leaves showed the highest total antioxidant capacity with values of 304.07 ± 1.11 mg AAE/mg, 191.85 ± 0.00 mg AAE/mg, and 173.09 ± 2.99 mg AAE/mg, respectively. Nevertheless, *Z. lotus* fruit extracts showed the lowest activity. Consequently, root and leaf extracts were significantly richer in phytochemicals and more active than the fruit extracts.

Removing water from the plant preserves the samples from deterioration and limits microbial multiplication [24]; however, it is crucial to determine possible effects of this drying process on the phytochemical compositions. The shade drying preserved the phytochemical composition of the extracts. In fact, for all the extracting solvents the dried roots showed significantly (*p* < 0.05) higher values of phenolic, flavonoids, and tannins than the fresh ones. As explained by Esparza-Martínez et al. [25], the drying process can ameliorate the cellular structure degradation leading to a better release of phenolic compounds. Nevertheless, for the leaves, no significant difference was observed in the phytochemical composition between the dried and fresh samples: the total phenolic composition might vary after a drying process depending on the plant tissue as well as the phenolic compound location in the cell [26].

Comparing the antioxidant activity of the dried samples to the fresh ones (Figure 2), the drying process significantly improved antioxidant capacity. Olufunmilayo et al. [27] attributed this difference to the richness of the dried samples in respect to fresh samples.

Exploring the chemical composition of the DR-DE extract, three compounds were identified (Table 5). The major compound was the tetradecanoic acid, ethyl ester (72.8%) belonging to the fatty acid ester class, known for its high hydrophobicity, and considered as a relatively neutral molecule used as a flavoring agent. This compound was previously identified in the fruit *n*-hexane fraction of *Z. lotus* [28]. The second major compound was the 13-epimanool (20.5%), a labdane-type diterpene. None of these compounds was previously identified in *Z. lotus* extracts or previously showed a cytotoxic potential.

The GC-MS/GC-FID analysis of DR-PEE (Table 5), revealed 6 compounds and palmitic acid (*n*-hexadecanoic acid), identified as the major compound (90.6%) of this extract, was reported as a potent cytotoxic agent against HCT-116 cells [29] and the human leukemic cells [30]. It has been also proved to be an apoptotic cell death inducer in human leukemic cell line MOLT-4. Hence, palmitic acid was suggested as an effective composite of anticancer remedies [30]. Additionally, 13-epimanool (0.8%) was detected as the common compound between the two active extracts, DR-DE and DR-PEE.

DR-PEE and DR-DE extracts were subjected to fractionation using the Thin Layer Chromatography (TLC) technique. Nine spots were isolated from the DR-PEE extract and six spots from the DR-DE, respectively named from the bottom E0 to E8 and D0 to D5.

Each isolated fraction was tested to evaluate its cytotoxic activity for 24 h. The spots named E6 (EC_50_ = 28.378 ± 0.47 µg/mL) and D4 (EC_50_ = 29.076 ± 1.39 µg/mL) were selected as the active fractions from the DR-PEE and DR-DE, respectively.

GC-MS/GC-FID analysis of the spots identified a unique compound 13-epimanool (100.0%) from the DR-PEE and two compounds from the DR-DE, 13-epimanool (85.1%) and ethyl tridecanoate (14.9%). Hence, 13-epimanool can be suggested to be responsible for the observed cytotoxic activity. Comparing the extracts to their active fractions, the activity of E6 (100% 13-epimanool) was approximately six times higher than the original extract DR-PEE. Consequently, 13-epimanool can be considered for the first time, to the best of our knowledge, as a potent cytotoxic compound expressing an affective antiproliferative activity. This gives the perspective to a deep study of this compound, extending from its isolation and purification to the determination of its mechanism of action.

*Z. lotus* extracts were tested to verify a possible cytotoxic activity on the SH-SY5Y human cell line and MTT assays were carried out. According to EC_50_ values on SH-SY5Y after 24 and 48 h of treatments, the dried root extracts of petroleum ether (DR-PEE) and dichloromethane (DR-DE) were selected and subsequent investigations were pursued. As reported by Rached et al. [31], the root barks are always expressing a potent cytotoxic effect. Consistent cytotoxic activities were also observed by testing the root bark extracts on hepatocellular HepG2 (48.3 µg/mL), breast MCF-7 (74 µg/mL) and cervical HeLa (69 µg/mL) [31].

The evaluation of the antiproliferative capacities of both extracts on neuroblastoma SH-SY5Y cells were reported in Table 5.

SH-SY5Y cells were more sensitive to DR-DE extract treatment in respect to the DR-PEE extract. In fact, after 24 h of treatment, the DR-PEE exhibited an EC_50_ of 16.148 ± 0.93 µg/mL regarding an EC_50_ of 184.413 ± 4.77 µg/mL for the DR-PEE. A comparative study investigating the *Neurolaena lobata* extracts, obtained by the increasing polarity extraction, reported the dichloromethane extract as the strongest antiproliferative agent against the human and murine anaplastic large cell lymphoma cell lines [32].

Furthermore, the evaluated activity of both samples showed time-dependent results since increasing the period of treatment to 48 h; EC_50_ values of 7.341 ± 1.98 µg/mL for DR-DE and 20.941 ± 1.16 µg/mL for DR-PEE were observed.

## 4. Materials and Methods

### 4.1. Preparation of Samples and Extracts

**Figure 3:**
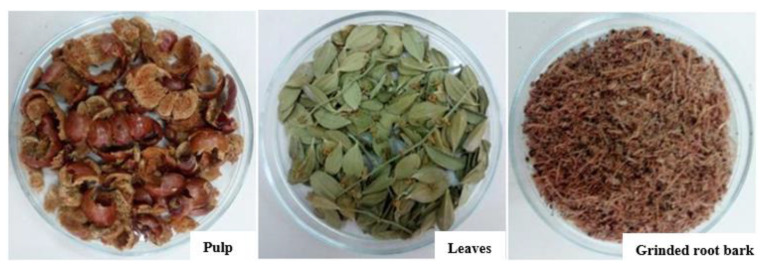
Different parts of *Ziziphus lotus.*

*Z. lotus* samples (leaves, fruits and roots) were collected in July 2017 from Oudhref-Gabes Region (South of Tunisia) (Figure 3). A part of the samples was cleaned and stored at 4 °C until use and the other part was shade-dried for two weeks at room temperature and stored in the absence of light and under dry conditions until use.

**Figure 4:**
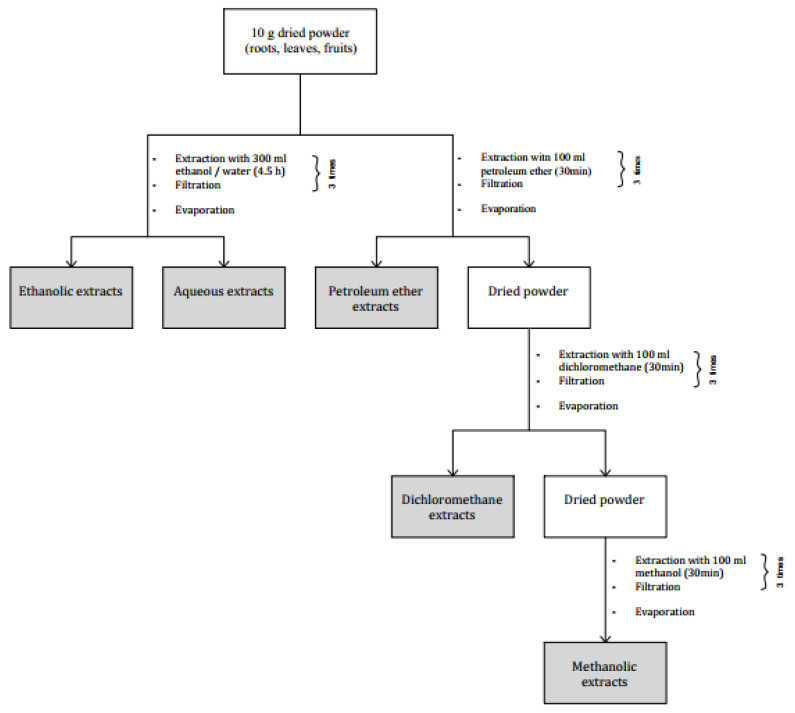
Preparation process of *Z. lotus* extracts.

For both fresh and dried plant material organ (leaves, fruits, and roots), two extraction methods were processed: (1) Powdered plant material was successively extracted with increasing polarity solvents, namely petroleum ether, dichloromethane, and methanol. Starting with petroleum ether, 10 g of powder was macerated 3 times in 100 mL of the solvent for 30 min under constant agitation at room temperature. After each maceration, the obtained mixture was filtered using a filter paper. Then, the obtained powder was extracted by the subsequent solvent using the same protocol as for petroleum ether. (2) Plant material was extracted independently with two green solvents, namely ethanol and water. Next, 10 g of the powder was macerated in 300 mL of solvent during 90 min under constant agitation at room temperature. Then samples were filtrated and evaporated by a rotary vacuum evaporator at 35 °C to remove the solvent (Figure 4).

The extraction yields were calculated:

(1) $$\mathrm{Yield}\;\mathrm{percentage}\;\left(\%\right)=\frac{w}{W}\times 100$$

w: the weight of residue in grams;
W: the weight of dried plant material in grams.

### 4.2. Total Phenolic Content

To determine phenolic content the Folin-Ciocalteu method was used [26]. A total of 100 µL of the diluted extract in DMSO was mixed with 500 µL of Folin Ciocalteu reagent (0.2 N). The mixture was kept in obscurity for 5 min at room temperature. Then, 400 µL of sodium carbonate solution (75 g/L, in water) was added; and after 30 min of incubation in the darkness, the absorbance was measured at 765 nm.

To plot a calibration curve, the gallic acid was used as standard and the amounts of phenolic content were expressed in milligrams of Gallic Acid Equivalents (GAE) per gram of Dry Weight (mg GAE/g DW). The measurements were carried out in triplicate.

### 4.3. Total Flavonoid Content

The evaluation of the flavonoid content was performed as outlined by Yahyaoui et al. [32] with some modifications. In brief, 250 µL of the solubilized extract was diluted in 2 mL of distilled water and mixed with 75 μL of a 15% sodium nitrite solution (NaNO_2_). After 6 min, 75 µL of 10% aluminum chloride solution (AlCl_3_) was added. After 6 min, 2 mL of sodium hydroxide solution (NaOH) (4%) was supplemented with a 100 µL of distilled water. After 15 min of incubation, the absorbance was measured at 510 nm.

The results were represented as milligrams of Quercetin Equivalent per gram of Dry Weight (mg QE/g DW) from the calibration relationship. The measurements were realized in triplicate.

### 4.4. Tannin Content

Tannins were estimated referring to the method cited by Ghazouani et al. [26], adapting some modifications. In an ice bath, 350 µL of each sample was mixed with 700 µL of vanillin (1% in 7 M H_2_SO_4_). After an incubation period of 15 min at 25 °C, the absorbance was measured at 500 nm. Results were expressed as mg of catechin equivalent per gram dry weight.

### 4.5. DPPH• Scavenging Activity

The antioxidant scavenging activity of *Z. lotus* extracts was evaluated using the 1,1-diphenyl-2-picrylhydrazyl (DPPH•) free radical method [26]. From each sample various dilutions were prepared, then a volume of 100 μL was mixed with 900 μL of freshly prepared methanolic DPPH• solution. After an incubation duration of 30 min in the darkness, the absorbance was measured at 520 nm using an UV-vis spectrophotometer.

Thus, the free radical-scavenging activity was expressed as the inhibition percentage defined as follow:

(2) % inhibition=100×(A(blank)−A(sample))/A(blank)

A (blank): the absorbance of the prepared DPPH• solution without the sample extract;
A (sample): the absorbance of the sample after the reaction with the DPPH• solution.

The DPPH• radical scavenging activity was expressed as the IC_50_ (mg/L), defined as the concentration of the test material able to reduce 50% of the initial DPPH• solution concentration. The IC_50s_ were graphically calculated using the linear regressions of the plotted lines; from the curves: %inhibition=f(treated extract concentrations).

Ascorbic acid was used as a standard. Each measurement was performed in triplicate.

### 4.6. ABTS^•+^ Scavenging Activity

The ABTS^•+^ (2,2’-azinobis-3-ethylbenzothiazoline- 6-sulphonate) scavenging activity of *Z. lotus* samples was assessed by referring to the experimental protocol described by Yahyaoui et al. [33]. Starting through the preparation of the 2, 2′-Azinobis-3-ethylbenzothiazoline-6-sulfonate by mixing the ABTS^•^^+^ aqueous solution (7 mM) with the potassium persulfate (2.5 mM) dissolved in water in the ratio of 1:1. The solution was used after 16 h of agitation in the darkness at room temperature. An amount of 100 µL of the sample was added to 900 µL of the diluted ABTS^•+^ solution. The absorbance was read at 734 nm after 6 min of incubation in the dark. The ABTS^•+^ radical scavenging activity was represented by the IC_50_ values (mg/L) estimated as the concentration expected to scavenge 50% of ABTS^•+^ radicals. The capacity of free radical scavenging IC_50_ was determined using the equation above using the DPPH• method. Measurements were performed in triplicate.

### 4.7. Total Antioxidant Capacity (TAC)

To determine the total antioxidant capacity of the samples, the phosphomolybdenum assay was processed [34]. The mixture of 0.1 mL of extract (0.5 mg/mL) and 1 mL of the reagent solution (0.6 M sulphuric acid, 28 mM sodium phosphate and 4 mM ammonium molybdate) was incubated for 90 min at 95 °C. The absorbance was measured at 765 nm. Results are expressed as milligram of ascorbic acid equivalent per gram of dry weight.

### 4.8. Thin Layer Chromatography (TLC)

TLC was conducted in a silica gel plate (20 cm × 20 cm, ICN Adsorbentien) and spots consisting of 80 µL of extract were processed [18]. The mobile phase was a mixture of petroleum ether-ethanol (7:3) and the run was proceeded for 3 h. At the end of the run, the plate was dried, the separated spots were viewed by using UV light and their outline marked with a pencil. Spots of the same line were scraped from the plate and the collected powders were extracted with ethanol for 30 min. After 5 min of centrifugation at 2500 rpm, the pellets were discarded and the supernatants concentrated in an evaporator.

### 4.9. GC-MS/GC-FID Analysis

Chemical analysis of *Z. lotus* samples was performed by a TurboMass Clarus 500 GC-MS/GC-FID from Perkin Elmer instruments (Waltham, MA, USA) equipped with a Stabilwax fused-silica capillary column (Restek, Bellefonte, PA, USA) (60 m × 0.25 mm, 0.25 µm film thickness). The operating conditions used were as follows: GC oven temperature was kept at 60 °C for 5 min and programmed to 220 °C at a rate of 5 °C/min, and kept constant at 220 °C for 25 min. Helium was used as a carrier gas at a flow rate of 1 mL/min. Solvent delay was 0–2 min and scan time was 0.2 s. Mass range was from 30 to 350 m/z using electron-impact at 70 eV mode. An amount of ~2 µg of each *Z. lotus* extract was diluted in 1 mL of methanol and 1 μL of the solution was injected into the GC injector at a temperature of 280 °C. The analysis was repeated twice. Relative percentages for quantification of the components were calculated by electronic integration of the GC-FID peak areas. The identification of the constituents was made by comparing the obtained mass spectra for each component with those reported in mass spectra Nist and Willey libraries. Linear retention indices (LRI) of each compound were calculated using a mixture of aliphatic hydrocarbons (C8–C30, Ultrasci) injected directly into GC injector at the same temperature program reported above.

### 4.10. Cytotoxic Activity

#### 4.10.1. Cell Cultures

The evaluation of the cytotoxic activity was carried out using the stabilized human neuroblast cells (SH-SY5Y, ATCC^®^ CRL-2266™) purchased from the American Type Culture Collection (ATCC-CRL2266). Cells were cultured in 75 cm^2^ flasks in DMEM-F12 (Dulbecco’s Modified Eagle’s Medium: nutrient mixture F-12) culture medium supplemented with 10% fetal bovine serum (FBS), 1% glutamine, and 1% penicillin/streptomycin in a humidified incubator at 37 °C and under 5% CO_2_. When the cells reached confluence, they were transferred into new flasks at a ratio of 1:20. Every four days, the medium was changed.

#### 4.10.2. MTT

The MTT [3-(4,5-dimethyl-2-thiazolyl)-2,5-diphenyl-2H-tetrazolium bromide] assay was adopted to evaluate the cytotoxic activity of *Z. lotus* extracts [35]. Cells were seeded (2 × 10^5^ cells/mL) in a 96 wells plate and incubated for 24 h before being treated with extracts diluted in DMSO. Treatment of the SH-SY5Y cells were carried out with different dilutions of samples ranging from 200 µg/mL to 0.39 µg/mL. Vinblastine sulfate (Merck KGaA, Darmstadt, Germany) and DMSO were used as positive and solvent controls, respectively. After the required incubation time, treatments were removed and a fresh medium with 0.5 mg/mL MTT was added. Plates were incubated for 4 h at 37 °C. The generated formazan crystals were solubilized by adding DMSO and absorbance was measured at 590 nm using a microplate reader (SunRise, TECAN, Inc, Boston, MA, USA). The percentage of viability was calculated as below:

(3) %viable cells =100×[1−(A(treated)/A(control))]

A(treated): absorbance mean of the treated cells
A(control): absorbance mean of the untreated cells

The EC50 was determined by using the linear regressions of the plotted curves describing the relation: %survived cells=f(extract concentrations).

Each measurement was performed in triplicate.

### 4.11. Statistical Analysis

Triplicate analyses were carried out to present results in means ± standard deviation. To compare the means, one way and two ways ANOVA was used. The statistical significance level was set up at *p* < 0.05. SPSS Statistics 28 software (IBM Corp., Armonk, NY, USA) was used to analyze the data.

## 5. Conclusions

Organic extracts prepared from the different parts of *Z. lotus* (leaves, fruit, roots) using solvents with different polarities were the subject of a phytochemical screening. The methanolic extract of the dried roots was the richest one in phytoconstituents, reflecting potent antioxidant activity. The petroleum ether and dichloromethane extracts of the dried roots showed notable cytotoxic activity against the SH-SY5Y cell line. The fractionation and the identification of these nonpolar extracts showed their richness in the 13-epimanool compound. Such findings justify the traditional use of this plant and encourage the investigation of *Z. lotus* in further studies.

## Figures and Tables

**Table 1:** Yield of extracts of *Z. lotus* extracts.

Extracts		Extraction Yield (%)
**Roots**	PEE	0.50
	DE	1.30
	ME	29.80
	EE	5.58
	AE	7.07
**Leaves**	PEE	4.20
	DE	2.80
	ME	15.10
	EE	9.23
	AE	12.97
**Fruits**	ME	25.30
	AE	48.00

PEE: Petroleum Ether Extract; DE: Dichloromethane Extract; ME: Methanol Extract; EE: Ethanol Extract; AE: Aqueous Extract

**Table 2:** Chemical composition of *Z. lotus* extracts.

Samples		TPC (mg GAE/g DW)	TFC (mg QE/g DW)	Tannins (mg CE /g DW)
**Roots**	PEE	39.22 ± 0.62 ^a^	19.63 ± 0.12 ^a^	9.14 ± 0.90 ^a^
	DE	30.15 ± 0.13 ^a^	18.50 ± 0.88 ^a^	6.90 ± 1.41 ^a^
	ME	186.44 ± 0.26 ^b^	102.50 ± 3.53 ^b^	60.71 ± 2.20 ^b^
	EE	100.13 ± 4.02 ^c^	41.25 ± 0.63 ^c^	27.70 ± 0.57 ^b^
	AE	146.06 ± 2.50 ^d^	36.70 ± 0.72 ^a^	31.86 ± 1.49 ^b^
Leaves	PEE	12.81 ± 0.10 ^e^	3.06 ± 0.12 ^d^	2.50 ± 0.37 ^c^
	DE	11.16 ± 0.45 ^e^	3.44 ± 0.06 ^d^	3.44 ± 0.47 ^c^
	ME	171.99 ± 1.14 ^f^	21.35 ± 1.19 ^e^	7.41 ± 0.68 ^d^
	EE	41.70 ± 0.70 ^g^	28.54 ± 1.89 ^f^	1.66 ± 0.09 ^d^
	AE	109.87 ± 2.07 ^h^	17.10 ± 1.30 ^d^	9.54 ± 0.26 ^d^
Fruits	ME	26.12 ± 0.73 ^i^	0.75 ± 0.13 ^g^	1.00 ± 0.170 ^e^
	AE	82.12 ± 1.70 ^j^	13.40 ± 0.72 ^h^	1.02 ± 0.10 ^e^

TPC: Total Phenolics content; TFC: Total Flavonoids content; GAE: Gallic Acid Equivalent; QE: Quercetin Equivalent; CE: Catechin Equivalent; PEE: Petroleum Ether Extract; DE: Dichloromethane extract; ME: Methanol extract; EE: Ethanol Extract; AE: Aqueous Extract. Lowercase letters represent Tukey’s Test comparison. Means within a column row with different letters were significantly different (*p* < 0.05).

**Table 3:** Antioxidant activities (IC_50_ mg/L) of *Z. lotus* extracts.

Samples		Antioxidant Assay		
		ABTS^•^^+^ IC_50_ (mg/L)	DPPH• IC_50_ (mg/L)	TAC (mg AAE/mg Extract)
Roots	PEE	14.76 ± 0.02 ^a^	101.06 ± 0.40 ^a^	105.56 ± 0.37 ^a^
	DE	136.58 ± 0.41 ^b^	192.33 ± 0.60 ^b^	91.11 ± 2.20 ^a^
	ME	14.31 ± 0.13 ^c^	18.03 ± 0.61 ^c^	304.07 ± 1.11 ^b^
	EE	27.42 ± 0.32 ^d^	39.50 ± 0.49 ^d^	167.41 ± 7.40 ^c^
	AE	8.96 ± 0.38 ^e^	16.46 ± 0.60 ^e^	191.85 ± 0.00 ^d^
Leaves	PEE	28.98 ± 0.06 ^f^	NA	NA
	DE	29.51 ± 1.23 ^g^	NA	154.44 ± 6.20 ^e^
	ME	23.48 ± 0.63 ^h^	33.66 ± 0.11 ^f^	142.47 ± 0.85 ^f^
	EE	249.37 ± 1.26 ^i^	375.50 ± 1.50 ^g^	173.09 ± 2.99 ^g^
	AE	29.01 ± 0.44 ^j^	64.80 ± 0.36 ^h^	99.26 ± 4.62 ^h^
Fruits	ME	173.93 ± 0.88 ^k^	343.00 ± 1.32 ^i^	26.42 ± 2.26 ^i^
	AE	342.25 ± 1.25 ^l^	383.33 ± 0.29 ^j^	40.74 ± 3.39 ^j^

ABTS^•^^+^: 2, 2′-azinobis-3-ethylbenzothiazoline-6-sulfonate; DPPH•: 1, 1-diphenyl-2-picrylhydrazyl; TAC: Total Antioxidant Activity; AAE: Ascorbic Acid Equivalents; PEE: Petroleum Ether Extract; DE: Dichloromethane Extract; ME: Methanol Extract; EE: Ethanol Extract; AE: Aqueous Extract. Lowercase letters represent Tukey’s Test comparison. Means within a column row with different letters were significantly different (*p* < 0.05); NA: Not Active. Ascorbic acid (IC_50_ = 2.74 ± 0.02 and 4.00 ± 0.00 mg/L for ABTS^•^^+^ and DPPH•, respectively) was used as antioxidant of reference to compare results.

**Table 4:** MTT assay on SH-SY5Y cells. IC_50_ of *Z. lotus* DR-PEE and DR-DE extracts.

	DR-PEE		DR-DE	
	After 24 h of Treatment	After 48 h of Treatment	After 24 h of Treatment	After 48 h of Treatment
IC_50_ (µg/mL)	184.413 ± 4.77	20.941 ± 1.16	16.148 ± 0.93	7.341 ± 1.98

**Table 5:** Chemical composition of *Z. lotus* active extracts and fractions.

Component	LRI ^1^	LRI ^2^	DR-PEE (%)	DR-DE (%)	^A^ Fraction (%)	^B^ Fraction (%)
Ethyl tridecanoate	1944	1943	0.2	6.7	-	14.9
2-pentadecanone	2026	2028	0.8	-	-	-
Tetradecanoic acid, ethyl ester	2055	2059	-	72.8	-	-
Pentadecanoic acid, ethyl ester	2178	2179	2.2	-	-	-
13-epimanool	2670	2676 *	0.8	20.5	100.0	85.1
Tetradecanoic acid	2680	2679	5.4	-	-	-
n-hexadecanoic acid	2943	2946	90.6	-	-	-
Total			100.0	100.0	100.0	100.0

^1^ Linear Retention indices measured on polar column; ^2^ Linear Retention indices from literature; * Normal alkane RI; ^A^ DR-PEE active fraction, ^B^ DR-DE active fraction; DR-PEE: Dried Root-Petroleum Ether Extract; DR-DE: Dried Root-Dichloromethane extract.

## Data Availability

All generated data are included in this article.
